# Mediation of academic self-efficacy between emotional intelligence and academic engagement in physical education undergraduate students

**DOI:** 10.3389/fpsyg.2023.1178500

**Published:** 2023-07-14

**Authors:** Raúl Baños, Juan José Calleja-Núñez, Roberto Espinoza-Gutiérrez, Antonio Granero-Gallegos

**Affiliations:** ^1^Faculty of Sport, Autonomous University of Baja California, Tijuana, Mexico; ^2^Department of Musical, Plastic and Corporal Expression, Faculty of Education Sciences, University of Granada, Ceuta, Spain; ^3^Department of Education, University of Almeria, Almeria, Spain; ^4^Health Research Centre, University of Almeria, Almeria, Spain

**Keywords:** engagement disaffection, emotional disaffection, emotional repair, emotional clarity, university

## Abstract

The aim of this study was to analyze academic self-efficacy as a mediator between emotional intelligence and academic engagement. A non-experimental, cross-sectional, correlational-causal study was designed in which 1,164 Mexican students participated (*M*_age_ = 21.21; *SD* = 3.26) (30.0% female; 69.6% male; 0.4% other). The scales of emotional intelligence, academic self-efficacy and academic engagement were used, and a structural equation analysis with latent variables was conducted. The results obtained demonstrate that emotional clarity and repair have a positive and direct effect on academic self-efficacy. In addition, emotional repair predicts behavioral and emotional engagement. It was also found that academic self-efficacy is an excellent mediator between emotional clarity and repair, and the dimensions of academic engagement, as it substantially improves behavioral and emotional engagement while decreasing behavioral and emotional disaffection.

## 1. Introduction

The university stage is a challenging period for students (Özhan and Boyaci, 2018; Asikainen et al., 2020) and is considered a major turning point in the lives of individuals (Bulfone et al., 2020). It is an important process in which students must focus their efforts on their own academic development in order to shape their personal and professional future. Understood that this drives university students to envision a series of expectations of success at the academic and social levels (Vizoso et al., 2019), it is paramount to raise awareness of the physical, mental and organizational factors that might impact the students’ success and reduce their potential negative effects (Özhan, 2021). But adding academic and family pressure on top of these expectations may lead to severe symptoms of psychological distress (Özhan and Boyaci, 2018), which can increase the likelihood of experiencing academic burnout and other outcomes such as school desertion (Boyaci and Özhan, 2018).

Preventing academic burnout and desertion is a problem that must be addressed in university teaching (Özhan, 2021), but this situation is even more severe in the Mexican context (Álvarez-Pérez and López-Aguilar, 2021). To counteract these negative effects on university students, several studies (e.g., Jeon et al., 2022) have highlighted the importance of devising learning strategies that foster academic engagement, as this is a significant component in preventing academic dropout. It is essential that students express engagement with their studies since it has been proven that academic engagement not only increases their probabilities of successfully completing their studies (Gao et al., 2020; Kim and Kim, 2021) but also improves their learning during corporate internships (Hong et al., 2021). In this line, several studies have underscored the importance of variables such as academic self-efficacy (Oriol-Granado et al., 2017) and emotional intelligence (Usán-Supervía et al., 2019) to improve academic engagement. However, studies that have analyzed the interaction of these three variables in the university context are scarce, and the ones that we identified have only surveyed students from Spain (Bonilla-Yucailla et al., 2022; Pérez-González et al., 2022). For these reasons, and because academic dropout rates in Mexico are truly worrying (Álvarez-Pérez and López-Aguilar, 2021; Vanegas et al., 2022), we consider it necessary to further delve into the interaction between these three variables in Mexican university students.

### 1.1. Academic engagement

Academic engagement has been assessed according to different theoretical standpoints (Alrashidi et al., 2016). On the one hand, one may refer to Fredicks’ model Fredricks et al. (2004), also known as the North American model, composed of three dimensions that measure academic engagement from a positive approach (i.e., behavioral, emotional and cognitive). Similarly, the model proposed by Schaufeli et al. (2002), known as the European model, measures academic engagement positively but using three other dimensions (i.e., absorption, vigor and dedication). However, Skinner et al. (2008) proposed that academic engagement should be measured from both a positive (i.e., engagement) and a negative (i.e., disaffection) perspective, each composed of two dimensions: cognitive and emotional. Thus, academic engagement would encompass four factors in total: behavioral engagement (i.e., persistence, attention and effort during the onset and execution of academic activities; Sinval et al., 2021), emotional engagement (i.e., the students’ positive and negative emotional responses to the learning process and class activities; Manwaring et al., 2017), behavioral disaffection (i.e., passivity and low student participation; Skinner et al., 2008) and emotional disaffection (i.e., boredom, anxiety and frustration experienced in the classroom; Skinner et al., 2008). We consider this theory to be of interest for our research, as it analyzes engagement from two standpoints, i.e., the positive and the negative.

Different studies (Liu et al., 2018; Zhen et al., 2020; Kuo et al., 2021) have highlighted the importance of academic self-efficacy on academic engagement after having analyzed the North American model by Fredricks et al. (2004). Other works (Azila-Gbettor and Abiemo, 2020; Zhao et al., 2021; Cai et al., 2022), following the European model by Schaufeli et al. (2002), have found that academic self-efficacy is a predictor of academic engagement. As can be seen, the scientific literature has already attested that academic self-efficacy predicts academic engagement, however, we are not aware of any studies that have delved into this relationship by having analyzed academic engagement from both positive and negative approaches. Moreover, research works that analyze the relationship between academic engagement and academic self-efficacy are virtually non-existent in the Mexican context. Therefore, and considering that academic self-efficacy positively predicts academic engagement, it is convenient to study how it relates to academic disaffection.

### 1.2. Academic self-efficacy

The theoretical construct of academic self-efficacy stems from the Social Cognitive Theory (Bandura, 1997) and is defined as the assumptions that students have about their own capabilities to organize and carry out the activities that are necessary to attain previously envisioned educational expectations (Gutiérrez and Tomás, 2019). Academic self-efficacy has a direct effect on satisfaction and the continuity of studies, thus helping prevent university students from dropping out (Lent et al., 2017). In this sense, students with high academic self-efficacy work harder, use more effective methods to deal with academic difficulties, are more willing to participate in learning activities, and have better performance compared to students with low academic self-efficacy (Fernandez-Rio et al., 2017; Gebauer et al., 2020). Hence, this variable (i.e., academic self-efficacy) relates to the students’ capabilities to identify opportunities and drawbacks in the environment, without prejudice to their engagement and motivation (Oriol-Granado et al., 2017). To our knowledge, although several studies have related academic self-efficacy to emotional intelligence (Bidhendi et al., 2018; Saeed and Ahmad, 2020; Pérez-González et al., 2022), only one study has analyzed the relationship between academic self-efficacy and emotional intelligence using structural equation modeling (SEM; Bonilla-Yucailla et al., 2022). These authors found that emotional intelligence positively and significantly predicts academic self-efficacy, with academic engagement playing a mediating role. However, said study only measured academic engagement from a positive perspective, so we consider it would be helpful to analyze this relationship taking the two possible academic engagement perspectives into consideration, i.e., positive and negative. In addition, given that the scientific literature fosters the role of academic self-efficacy as a predictor of academic engagement (Casas and Blanco-Blanco, 2017; Oriol-Granado et al., 2017; She et al., 2021; Akanni, 2022; Azila-Gbettor et al., 2022), we consider it interesting to study the relationships between these three constructs while analyzing the mediating role of academic self-efficacy between emotional intelligence and academic engagement.

### 1.3. Emotional intelligence

A substantial body of literature has underscored the importance of emotional intelligence in the occurrence of positive emotional responses during the learning process (Thomas and Heath, 2022). In the university context, emotional intelligence has stood out as an adequate tool for coping with stressful situations, and for achieving a successful academic performance and emotional well-being (Parhiala et al., 2018; Guil et al., 2021). Salovey et al. (1995) suggest that emotional intelligence is composed of three dimensions and that it can be defined as an individual’s capacity to address (emotional attention), understand (emotional clarity), and alter (emotional repair) their own emotional states. Different studies have emphasized the important role of Salovey’s theory in terms of engagement with learning, satisfaction, and academic performance (Baños et al., 2019; Fernández-Lasarte and Axpe Sáez, 2019; Supervía et al., 2019).

Emotional intelligence (i.e., emotional attention, emotional clarity and emotional repair) has been recently related to the academic engagement of secondary (junior high) school students (Supervía et al., 2019; Supervía and Bordás, 2019; Usán-Supervía et al., 2019) using the European model by Schaufeli (2013). Similarly, emotional intelligence (albeit unidimensionally measured) has also been related to the academic engagement of university students (Thomas and Allen, 2020; Thomas and Heath, 2022) according to Skinner’s model Skinner et al. (2008), in which academic engagement is addressed from a positive and negative perspective. In fact, to our knowledge, these (Thomas and Allen, 2020; Thomas and Heath, 2022) are the only studies that have related emotional intelligence to Skinner’s model of academic engagement; said studies, however, did not measure emotional intelligence from the dimensions of attention, clarity and repair, but rather unidimensionally. These works pointed out the importance of considering the positive effect of emotional intelligence on behavioral engagement and emotional engagement. Conversely, students with high emotional intelligence relate negatively to behavioral disaffection and emotional disaffection (Thomas and Heath, 2022). As can be seen, scientific works that relate the dimensions of emotional intelligence (emotional attention, emotional clarity and emotional repair) to the academic engagement model by Skinner et al. (2008) are non-existent. Therefore, we consider it interesting to thoroughly analyze how these two constructs relate within the Mexican context.

### 1.4. This study

**Figure 1:**
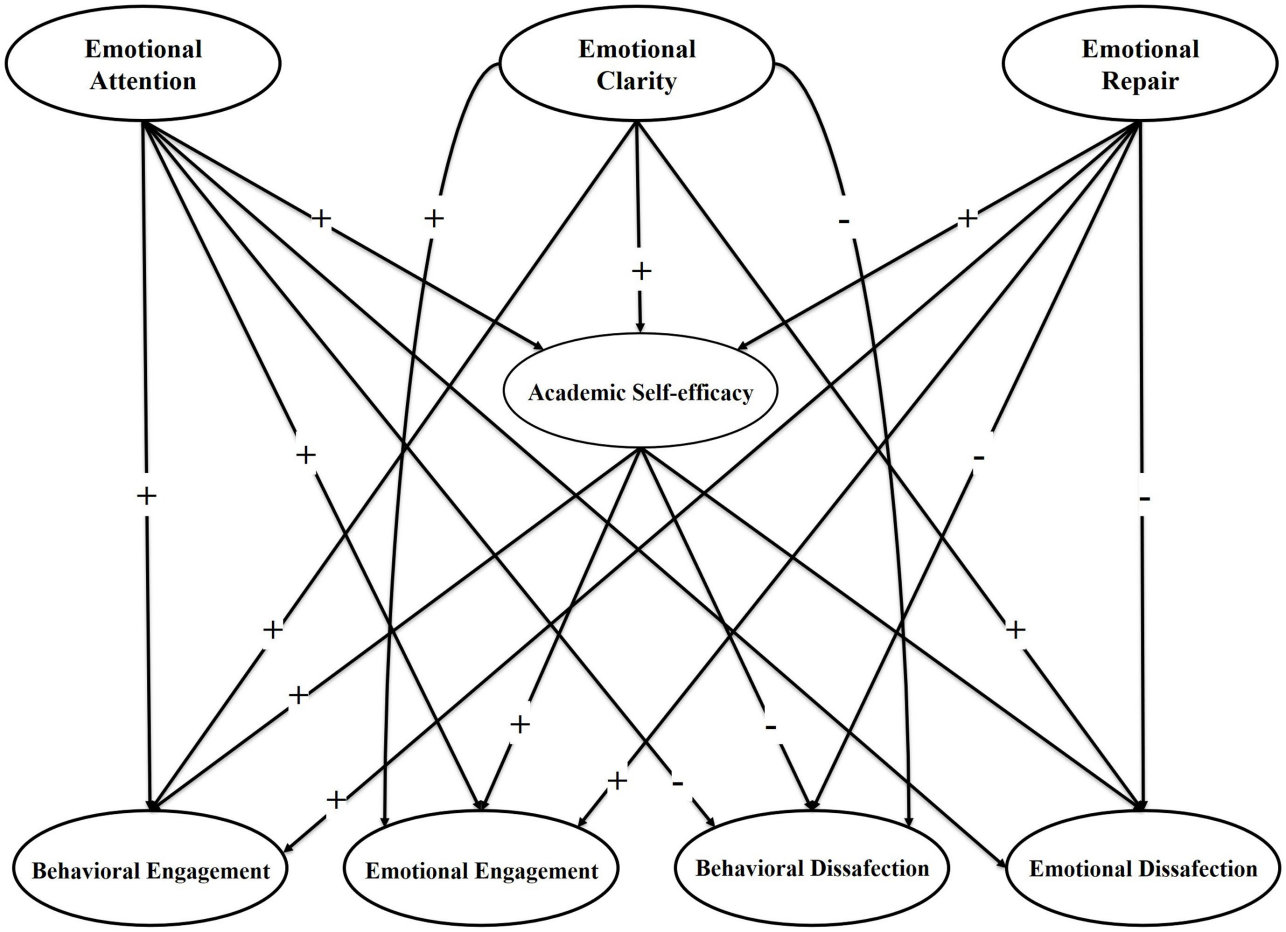
Modelo hipotetizado con las relaciones esperadas.

Having analyzed the scientific literature and observed the importance of academic engagement in the education of university students, we consider the predictive analysis of emotional intelligence and academic self-efficacy on academic engagement to be relevant, and that studying it should lead to learning improvements that benefit Mexican university students. In summary: on the one hand, even though studies have related academic self-efficacy to either the North American model (Liu et al., 2018; Zhen et al., 2020; Kuo et al., 2021) or the European model of academic engagement (Azila-Gbettor and Abiemo, 2020; Zhao et al., 2021; Cai et al., 2022), these have only analyzed academic engagement from a positive perspective, without delving into how academic self-efficacy relates to academic disaffection. On the other hand, while few studies are known to have analyzed emotional intelligence unidimensionally with academic engagement and disaffection (Thomas and Allen, 2020; Thomas and Heath, 2022), others have analyzed how the dimensions of emotional intelligence correlate according to the European model of academic engagement, i.e., without measuring academic disaffection (Supervía et al., 2019; Supervía and Bordás, 2019; Usán-Supervía et al., 2019. Furthermore, as far as we are aware, the analysis of the role of academic self-efficacy as a mediator between emotional intelligence and academic engagement and disaffection has not been addressed. Therefore, this study intends to be a relevant contribution to the understanding of the relationship between the dimensions of emotional intelligence, academic self-efficacy and academic engagement, especially by incorporating the variables of academic disaffection, given that studies that have taken academic disaffection into consideration are scarce both in the Mexican university context and worldwide. Figure 1 shows the hypothesized model of this research to examine the aforementioned relationships. Thus, the purpose of this research is to analyze the mediation of academic self-efficacy between emotional intelligence and academic engagement. The *Strengthening the Reporting of Observational Studies in Epidemiology* (STROBE) initiative (Von Elm et al., 2008) was used for the description of the study.

## 2. Materials and methods

### 2.1. Design and participants

The design of this research was descriptive, observational, cross-sectional and non-randomized. Participants study at either of the three campuses of the Faculty of Sport of the Autonomous University of Baja California, Mexico (i.e., Campus Tijuana, Campus Mexicali and Campus Ensenada). Inclusion criteria for participation in the study were the following: (i) to be enrolled in the Bachelor’s degree in Physical Activity and Sport at either of the three aforementioned campuses and attend classes regularly; (ii) to give their informed consent for data collection; (iii) to duly fill out the data collection form with the different scales. An *a priori* analysis of the necessary sampling size was conducted to provide an answer to the proposed objective, considering a structural equation model (SEM) composed of six latent variables and 49 observable variables. The analysis was conducted using the *Free Statistics Calculator* v.4.0 software (Soper, 2022) and a minimum of 1,164 participants was calculated to detect effect sizes (*f*^2^) = 0.166), with a statistical power of 0.99% and a significance level of α = 0.05. The total number of participants was 1,164 university students of the Bachelor’s degree in Physical Education (349 women, 810 men and 5 other) from the Tijuana (*n* = 577), Mexicali (*n* = 357) and Ensenada (*n* = 230) University Campuses. Students were aged between 17 and 50 years old (*M* = 21.21; *SD* = 3.26). It must be mentioned that 14 individuals did not give their consent to participate in the research and that 29 questionnaires were discarded because they were filled incorrectly. Lastly, there were no lost values in the responses included in the study.

### 2.2. Instruments

#### 2.2.1. Traid meta-mood scale-24

This study used the Mexican version by Valdivia Vázquez et al. (2015) adapted from the original by Salovey et al. (1995). The scale is composed of 28 items distributed across three dimensions: *emotional attention* (8 items; e.g., I pay close attention to my feelings. “*Presto mucha atención a mis sentimientos*”), *emotional clarity* (8 items; e.g., I frequently can define my feelings. “*Frecuentemente puedo definir mis sentimientos*”) and *emotional repair* (8 items; e.g., I try to have positive thoughts, even when I feel bad. “*Intento tener pensamientos positivos, aunque me sienta mal*”). Answers were collected using a Likert scale ranging from 1 (*totally disagree*) to 5 (*totally agree*). For this study, the CFA goodness-of-fit indices were acceptable: *χ*^2^/gL = 4.97, *p* < 0.001; CFI = 0.98; TLI = 0.97; RMSEA = 0.058 (90%CI = 0.048, 0.069; *p*_close_ = 0.089), SRMR = 0.037. Reached reliability was: emotional attention, ω = 0.86; emotional clarity, ω = 0.86; emotional repair, ω = 0.87.

#### 2.2.2. Academic self-efficacy

This study used the scale adapted to the Mexican context by Córdova (2019) based on the original by Palenzuela (1983). This instrument is composed of 13 items that measure academic self-efficacy unidimensionally (e.g., I believe I am capable of understanding a subject well. “*Pienso que tengo capacidad para comprender bien una materia*”). Answers were collected using a Likert scale ranging from 1 (*never*) to 4 (*always*). In this study, the CFA goodness-of-fit indices were acceptable: *χ*^2^/gL = 3.37, *p* < 0.001; CFI = 0.99; TLI = 0.99; RMSEA = 0.045 (90%CI = 0.028, 0.063; *p*_close_ = 0.643), SRMR = 0.016. Reached reliability was ω = 0.88.

#### 2.2.3. Academic engagement

This study used the Mexican adaptation by Rodríguez-Medellín et al. (2020) from the original scale by Chi et al. (2010). The scale contains 12 items grouped into four subscales of three items each: *emotional engagement* (e.g., Class contents are interesting. “*Es interesante el contenido que vemos en las clases*”), *behavioral engagement* (e.g., I try to do the most I can in classes. “*Trato de hacer lo más que puedo en las clases*”), *emotional disaffection* (e.g., I get stressed during classes. “*Me estreso en las clases*”) and *behavioral disaffection* (e.g., I do not do a lot of work during classes. “*No hago mucho trabajo en las clases*”). Answers were collected using a Likert scale ranging from 1 (*false*) to 5 (*true*). The CFA goodness-of-fit indices for this study were deemed acceptable: *χ*^2^/gL = 4.48, *p* < 0.001; CFI = 0.96; TLI = 0.94; RMSEA = 0.055 (90%CI = 0.047, 0.063; *p*_close_ = 0.167), SRMR = 0.036. Reached reliability was: behavioral engagement, ω = 0.69; emotional engagement, ω = 0.69; behavioral disaffection, ω = 0.57; emotional disaffection, ω = 0.71.

### 2.3. Procedure

The general director of the Faculty of Sport and the deputy directors of the three campuses (Tijuana, Mexicali and Ensenada) of the Autonomous University of Baja California were contacted to inform them of the purpose of the research and to request permission to apply the questionnaires. Upon granted authorization, an online questionnaire was administered in person in the institution’s computer room in March 2022. Participants were taught how to use the scales and informed about the importance of the research, that their answers were anonymous and would therefore not affect their scores, and that they could abandon the study at any time if they so desired. All participants included in the study gave their prior consent for their responses to be used. The research protocol was approved by the Bioethics Committee of the University of Almeria (Ref:UALBIO2020/019).

### 2.4. Statistical analysis

Descriptive statistics, the correlations among variables and McDonald’s omega (ω) coefficient (McDonald, 1970) were initially calculated using SPSS v.28 for each dimension, assuming that values >0.70 indicate adequate reliability (Viladrich et al., 2017). Main analyses were performed using AMOS v.26, and a two-step SEM with latent variables following Kline (2016) was calculated to evaluate the predictive relationships of the dimensions of emotional intelligence on the dimensions of academic engagement, analyzing the mediating role of academic self-efficacy. In the first step of the SEM, known as the measurement model, the robustness of the bidirectional relationships between the model variables was assessed. In the second step, the predictive effects between the variables were examined, with the SEM effects being controlled according to the gender and campus of origin of the students. Due to the violation of the multivariate normality assumption (Mardia’s coefficient = 138.61; *p* < 0.001), the analysis was conducted using the maximum likelihood estimation method and the 5,000-iteration bootstrapping procedure (Kline, 2016). The SEM were assessed with the following goodness-of-fit indices: values of the chi-square/degrees of freedom ratio (*χ*^2^/gL), Comparative Fit Index (CFI), Tucker–Lewis Index (TLI), Root Mean Square Error of Approximation (RMSEA) with a confidence interval of 90% (CI), and Standardized Root Mean Square (SRMR). For the *χ*^2^/gL ratio, values <2.0 or < 5.0 are, respectively, considered excellent (Tabachnick and Fidell, 2019) or acceptable (Hu and Bentler, 1999); for the CFI and the TLI, values >0.95 are deemed excellent, whereas the range between 0.90 and 0.95 is considered acceptable; for RMSEA and SRMR, values <0.06 are considered excellent (Hu and Bentler, 1999; Marsh et al., 2004). The internal consistency of each instrument was assessed using McDonald’s ω, considering that values >0.70 are deemed acceptable. In this study, three factors of academic engagement (i.e., behavioral engagement, emotional engagement, and behavioral disaffection) showed reliability values <0.70, however, according to Taylor et al. (2008), these can be considered marginally acceptable due to the small number of items (three) in each dimension.

## 3. Results

### 3.1. Resource identification initiative

Descriptive statistics and correlations between the different variables are shown in Table 1.

**Table 1:** Descriptive statistics and correlations among variables.

Variable	Range	M	SD	Q1	Q2	2	3	4	5	6	7	8
1. Emotional attention	1–5	3.46	1.10	−0.35	−0.41	0.21**	0.08**	0.04	0.13**	0.07*	−0.00	0.01
2. Emotional clarity	1–5	3.43	1.05	−0.30	−0.12	-	0.33**	0.31**	0.22**	0.18**	−0.09**	−0.13**
3. Emotional repair	1–5	3.76	1.03	−0.64	1.55		–	0.27**	0.23**	0.27**	−0.06*	−0.18**
4. Academic self-efficacy	1–4	3.06	0.59	−0.29	−0.41			–	0.43**	0.38**	−0.20**	−0.22**
5. Behavioral engagement	1–5	3.90	0.67	−0.35	−0.12				–	0.49**	−0.40**	−0.34**
6. Emotional engagement	1–5	4.24	0.67	−1.05	1.55					–	−0.19**	−0.47**
7. Behavioral disaffection	1–5	2.41	0.89	0.37	−0.30						–	0.41**
8. Emotional disaffection	1–5	1.90	0.78	1.05	1.45							–

**Correlation is significant at level 0.01; *Correlation is significant at level 0.05; M = Mean; SD=Standard Deviation; Q1 = Skewness; Q2 = Kurtosis; ɷ = McDonald’s Omega.

### 3.2. Main analysis

**Figure 2:**
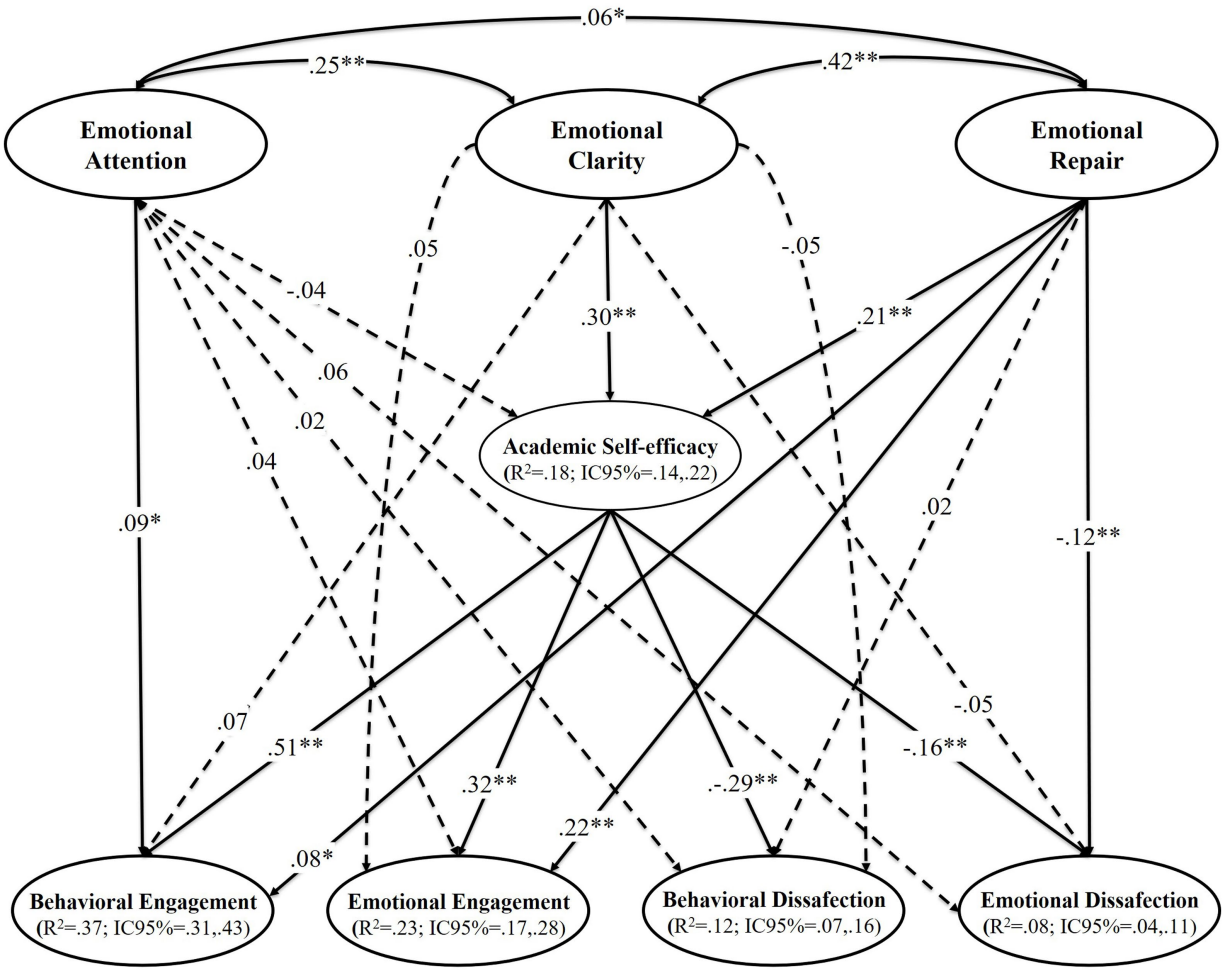
Predictive relationships of the emotional intelligence on academic engagement through the mediating role of the academic self-efficacy. **p < 0.01; *p < 0.05. *R*^2^ = Explained variance; CI=Confidence interval. The dashed lines represent non-significant relationships.

The SEM showed acceptable goodness-of-fit indices during step 1: *χ*^2^/gL = 2.71, *p* < 0.001; CFI = 0.96; TLI = 0.95; RMSEA = 0.038 (90%CI = 0.035;0.041; *p*_close_ = 0.999), SRMR = 0.039. During step 2, the SEM showed a similar and acceptable fit: *χ*^2^/gL = 2.71, *p* < 0.001; CFI = 0.96; TLI = 0.95; RMSEA = 0.038 (90%CI = 0.035;0.041; *p*_close_ = 0.999), SRMR = 0.039. The model was controlled by the sex and campus of origin variables and reached an explained variance of 37% for behavioral engagement, 23% for emotional engagement, 12% for behavioral disaffection, 8% for emotional disaffection and 18% for academic self-efficacy (Figure 2). The relationships among the dimensions of emotional intelligence (i.e., emotional attention, emotional clarity and emotional repair), academic self-efficacy and the four dimensions of academic engagement (i.e., behavioral engagement, emotional engagement, behavioral disaffection and emotional disaffection) can be attested in Figure 2 and Table 2.

**Table 2:** Estimación de parámetros estandarizados significativos y estadísticas del modelo de mediación.

	Independent variable	Dependent variable	Mediator	β	SE	95%CI	
						Inf	Sup
*Direct effects*							
	Emotional attention	Behavioral engagement		0.09*	0.04	0.03	0.16
	Emotional repair	Behavioral engagement		0.08*	0.04	0.02	0.15
	Emotional repair	Emotional engagement		0.22**	0.05	0.15	0.30
	Emotional repair	Emotional disaffection		−0.12**	0.04	−0.18	−0.04
	Emotional clarity	Academic self-efficacy		0.30**	0.04	0.23	0.37
	Emotional repair	Academic self-efficacy		0.21**	0.05	0.14	0.27
	Academic self-efficacy	Behavioral engagement		0.51**	0.04	0.45	0.57
	Academic self-efficacy	Emotional engagement		0.32**	0.05	0.25	0.40
	Academic self-efficacy	Behavioral disaffection		−0.29**	0.05	−0.36	−0.20
	Academic self-efficacy	Emotional disaffection		−0.16**	0.05	−0.23	−0.09
*Indirect effects*							
	Emotional clarity	Behavioral engagement	Academic self-efficacy	0.15**	0.02	0.12	0.19
	Emotional clarity	Emotional engagement	Academic self-efficacy	0.10**	0.02	0.07	0.13
	Emotional clarity	Behavioral disaffection	Academic self-efficacy	−0.09**	0.02	−0.12	−0.06
	Emotional clarity	Emotional disaffection	Academic self-efficacy	−0.05**	0.02	−0.08	−0.03
	Emotional repair	Behavioral engagement	Academic self-efficacy	0.11**	0.02	0.07	0.15
	Emotional repair	Emotional engagement	Academic self-efficacy	0.07**	0.02	0.04	0.01
	Emotional repair	Behavioral disaffection	Academic self-efficacy	−0.06**	0.02	−0.09	−0.04
	Emotional repair	Emotional disaffection	Academic self-efficacy	−0.03**	0.01	−0.06	−0.02
Total effects							
	Emotional clarity	Behavioral engagement		0.22**	0.05	0.15	0.32
	Emotional clarity	Emotional engagement		0.15**	0.05	0.08	0.23
	Emotional clarity	Behavioral disaffection		−0.14*	0.05	−0.22	−0.05
	Emotional clarity	Emotional disaffection		−0.10*	0.05	−0.18	−0.03
	Emotional repair	Behavioral engagement		0.19**	0.05	0.11	0.26
	Emotional repair	Emotional engagement		0.29**	0.04	0.22	0.36
	Emotional repair	Emotional disaffection		−0.15**	0.04	−0.22	−0.08

β, Estimation of standardized parameters; SE, standard error; 95% CI, 95% confidence interval; Inf, Inferior limit of 95% CI; Sup, Superior limit of 95% CI; **p < 0.01; *p < 0.05.

Figure 2 outlines the SEM and demonstrates that emotional clarity has a direct, positive, and significant relationship with academic self-efficacy. On the other hand, emotional repair correlates directly, positively, and significantly with emotional engagement and behavioral engagement, and negatively with emotional disaffection. Emotional attention shows a direct, positive and significant relationship only with behavioral engagement. Likewise, academic self-efficacy has a positive and significant direct effect on behavioral engagement and emotional engagement, and a negative and significant effect on behavioral disaffection and emotional disaffection. The mediating role of academic self-efficacy must be highlighted, as it indirectly, significantly and positively relates emotional clarity with behavioral engagement and emotional engagement, and significantly and negatively relates emotional clarity with behavioral disaffection and emotional disaffection. In addition, academic self-efficacy acts as a positive and significant mediating variable between emotional repair and behavioral engagement and between emotional repair and emotional engagement, and as a negative mediator between emotional repair and behavioral disaffection and between emotional repair and emotional disaffection. Lastly, the CI (95%) of *R*^2^ can be attested in Figure 2, thereby confirming that these can be considered ES measurements (Dominguez-Lara, 2017).

## 4. Discussion

The aim of this research was to analyze the role of academic self-efficacy as a mediator between emotional intelligence and academic engagement. The main results demonstrate the important role of academic self-efficacy since, on the one hand, emotional clarity is only related to the dimensions of academic engagement and disaffection through academic self-efficacy. On the other hand, it significantly increases the total effects of emotional repair on behavioral engagement, emotional engagement and emotional disaffection.

We are not aware of any research that has related the dimensions of emotional intelligence (i.e., emotional attention, emotional clarity and emotional repair) to academic engagement and disaffection (i.e., behavioral engagement, emotional engagement, behavioral disaffection, and emotional disaffection). Our work shows that emotional repair directly and positively predicts behavioral engagement, but especially emotional engagement. In contrast, emotional repair directly and negatively predicted emotional disaffection. These results follow the path of other studies carried out with university students where emotional intelligence (measured unidimensionally) predicts behavioral and emotional engagement positively, and behavioral and emotional disaffection negatively (Thomas and Allen, 2020; Thomas and Heath, 2022). Other studies conducted with middle school students (Supervía et al., 2019; Supervía and Bordás, 2019; Usán-Supervía et al., 2019) also obtained positive relationships for all dimensions of emotional intelligence (i.e., emotional attention, emotional clarity and emotional repair) according to the European model of academic engagement; however, these did not analyze academic disaffection. This positive relationship between emotional intelligence and academic engagement might stem from the fact that students with higher emotional intelligence are more likely to experience achievement-inducing emotions such as interest, enjoyment and enthusiasm, while being less likely to undergo negative emotions, e.g., boredom, anxiety and frustration (Pekrun et al., 2007). We consider this to be a contribution of our study to the scientific literature, given that the dimensions of emotional intelligence had not been previously analyzed according to the academic engagement and disengagement model.

In this study, emotional attention directly and positively predicted behavioral engagement only, and did not show any significant relationship with the indirect and total effects of the model. Other studies conducted with middle school students obtained similar results, in which emotional attention had a weak relationship with academic engagement (Supervía et al., 2019; Supervía and Bordás, 2019; Usán-Supervía et al., 2019). This weakness in the prediction of emotional attention could be due to the fact that, unlike emotional clarity and emotional repair (Salovey et al., 1995), this dimension does not have great inference in people’s behavior. However, we believe that the relationship between emotional intelligence according to Salovey et al. (1995) and academic engagement and disaffection should be further studied.

It is worth underscoring the important role of academic self-efficacy in the SEM proposed in this study, not only because it considerably increases the total effects of emotional repair, but also because, more importantly, emotional clarity only relates to academic engagement and disaffection through academic self-efficacy as a mediating variable. We are not aware of earlier studies that have analyzed the relationships among the models of the variables featured in this research. However, works using other theoretical constructs of emotional intelligence and academic engagement did find a positive correlation between emotional intelligence, academic self-efficacy and academic engagement (Bonilla-Yucailla et al., 2022; Pérez-González et al., 2022). Other studies also found that high emotional intelligence related to high academic self-efficacy (Bidhendi et al., 2018; Saeed and Ahmad, 2020; Pérez-González et al., 2022), and that academic self-efficacy did predict academic engagement (Casas and Blanco-Blanco, 2017; Oriol-Granado et al., 2017; She et al., 2021; Akanni, 2022; Azila-Gbettor et al., 2022). A potential explanation for this is that when students are more capable of understanding their emotions and dissipating feelings of frustration in their academic life by having different strategies at hand that help them approach class tasks efficiently, this can in turn help them increase their engagement with learning (Pekrun et al., 2007). Our study makes a relevant contribution to the scientific literature by highlighting the important roles of academic self-efficacy, clarity and emotional repair in increasing academic engagement. On the contrary, if emotional intelligence and academic self-efficacy are not developed in university students, they will see their academic disaffection increase, which relates to lower performance (Kim and Kim, 2021) and even academic dropout (Gao et al., 2020). However, since the scientific literature in this area is scarce, we consider that this relationship should be taken with caution, and we suggest conducting further research to analyze the relationships among the variables presented in this study.

Lastly, we will disclose a series of limitations and strengths observed in our study, as well as future research outlooks. The main limitations include: (i) the cross-sectional design of the study, which did not allow us to establish causal inferences; (ii) the potential social desirability bias due to the use of self-reporting, since participants may have exaggerated when filling out their form; (iii) there was no sample randomization. On the other hand, the strengths of this research should be highlighted: (i) the sample size of Mexican university students from the three campuses (Tijuana, Mexicali and Ensenada) enrolled in the Bachelor’s Degree in Physical Activity and Sport Sciences at the Autonomous University of Baja California; (ii) the subject matter is of great value and current relevance in educational research, as studies that relate emotional intelligence, academic self-efficacy and academic engagement are still scarce. We consider it necessary for researchers to delve further into the relationships between these variables using different research designs (e.g., experimental or longitudinal) to provide more evidence to help explain how the analyzed variables interrelate with one another. It would also be convenient for future researchers to work with students from other degree programs or to conduct cross-cultural research with other states in Mexico, or with other countries, analyzing potential differences. To conclude, due to the major role that teachers play in the academic engagement of university students (Moreno-Murcia and Corbí, 2021), especially considering the frustration of basic psychological needs and burnout endured by Mexican teachers (Delgado-Herrada et al., 2021; Luis Rojas-Solís et al., 2021), we consider it interesting for future researchers to analyze how teachers can influence the emotional intelligence, academic self-efficacy and academic engagement of university students.

## 5. Conclusion

In conclusion, it can be attested that emotional clarity and repair have a direct and positive effect on academic self-efficacy, as do emotional repair on behavioral and emotional engagement, and emotional attention on behavioral engagement. However, academic self-efficacy is an excellent mediator between emotional intelligence and the dimensions of academic engagement, as it substantially improves behavioral and emotional engagement while decreasing behavioral and emotional disaffection. Finally, teachers should present students with different learning strategies that teach them how to be efficient in their learning and to understand the feelings they experience, remediating potential negative emotions derived from frustrations or unattained achievements in order to face future academic situations.

### 5.1. Practical implications

The results of this research underscore the importance of emotional clarity, emotional repair and especially academic self-efficacy in the development of academic engagement in Mexican students. Therefore, those responsible for education matters should devise and establish psychoeducation groups that strengthen the academic self-efficacy of university students (Özhan, 2021). Accordingly, teachers should use different learning strategies to provide students with numerous learning tools and techniques, such as the ones proposed by different researchers (Baños et al., 2021, 2022; Gómez López et al., 2022; González-Fernández et al., 2022; Martín-Moya et al., 2022; Pérez-Suasnavas and Cela, 2022; Sánchez-Cabrero and Pericacho-Gómez, 2022;). In this way, students will be able to try and experience different educational resources, analyzing which of them will be more effective in their learning. To this end, the psycho-pedagogical areas can organize workshops for teachers to support the teaching and learning processes by creating positive classroom environments, acknowledging the motivations of each student and fostering academic engagement in them, understanding that, if they learn how to be efficient, they will obtain good results (Oriol-Granado et al., 2017; Pan, 2022). In other words, when university students develop academic self-efficacy, they will not only see their academic engagement improve (Álvarez-Pérez et al., 2021), but also feel efficient during their internships and in their future jobs (Martínez-Martínez and Ventura, 2020; Hong et al., 2021).

As a final consideration, students should be first taught how to be efficient not at the university stage, but in earlier years. Significant time must be allocated to inform middle and high school students about career guidance and job opportunities, and to teach them how to be efficient. However, students perceive that some schools do not prepare them adequately for higher education or do not provide them with the skills and information needed to thrive in higher education (Thomas and Maree, 2022).

## Data availability statement

The raw data supporting the conclusions of this article will be made available by the authors, without undue reservation.

## Ethics statement

The studies involving human participants were reviewed and approved by the University of Almería (Ref: UALBIO2023/001). The patients/participants provided their written informed consent to participate in this study.

## Author contributions

JJC-N, RB, and AG-G conceived the hypothesis of this study. JJC-N and RE-G participated in data collection. RB and AG-G analyzed the data. RB, JJC-N, AG-G, and RE-G wrote the manuscript with the most significant input from RB. All authors contributed to the data interpretation of statistical analysis and read and approved the final manuscript.

## Funding

This article was carried out related to a research stay of AG-G at the Autonomous University Baja California (from 01 February 2023 to 16 Mach 2023) with JJC-N.

## Conflict of interest

The authors declare that the research was conducted in the absence of any commercial or financial relationships that could be construed as a potential conflict of interest.

## Publisher’s note

All claims expressed in this article are solely those of the authors and do not necessarily represent those of their affiliated organizations, or those of the publisher, the editors and the reviewers. Any product that may be evaluated in this article, or claim that may be made by its manufacturer, is not guaranteed or endorsed by the publisher.
